# Hyperoxia affects peripheral tissue microcirculation in patients with pulmonary arterial hypertension

**DOI:** 10.1186/cc10813

**Published:** 2012-03-20

**Authors:** S Dimopoulos, G Tzanis, C Manetos, A Tasoulis, A Mpouchla, E Tseliou, I Vasileiadis, N Diakos, J Terrovitis, S Nanas

**Affiliations:** 1 University of Athens, Greece

## Introduction

Pulmonary microcirculation abnormalities play a central role in pulmonary arterial hypertension (PAH) pathophysiology. We hypothesized that PAH patients also have systemic muscle microcirculation alterations compared to healthy subjects. The aim of this study was to investigate peripheral muscle microcirculation by near-infrared spectroscopy (NIRS) in PAH patients and to test the effects of hyperoxia into their tissue microcirculation.

## Methods

Eight PAH patients and eight healthy subjects matched for age, gender and body mass index underwent NIRS evaluation. Tissue O_2 _saturation (StO_2_, %), defined as the percentage of hemoglobin saturation in the microvasculature compartments, was measured on the thenar muscle. Subsequently, the 3-minute brachial artery occlusion technique was applied before, during, and after 15 minutes of 100% of O_2_-breathing. Main measurements included the oxygen consumption rate (OCR, %/minute), the reactive hyperemia time (RHT, minutes), and the time needed for StO_2 _to reach its baseline values after the release of the occlusion.

## Results

PAH patients had a significantly lower resting StO_2 _(65.8 ± 14.9 vs. 82.1 ± 4.0, *P *= 0.01), a lower OCR (35.3 ± 9.1 vs. 43.4 ± 19.7) and a higher RHT (3.0 ± 0.6 vs. 2.0 ± 0.3, *P *< 0.001) compared to controls. Hyperoxic breathing increased StO_2 _(65.8 ± 14.9 to 71.4 ± 14.5, *P *< 0.05) in PAH patients, while OCR was reduced (35.3 ± 9.1 to 25.1 ± 6.6, *P *< 0.05) and RHT was further increased (3.0 ± 0.6 to 4.2 ± 0.7, *P *< 0.01).

## Conclusion

PAH patients present a significant impairment of peripheral tissue microcirculation as assessed by the NIRS occlusion technique. Acute hyperoxic breathing affects peripheral microcirculatory function in PAH patients, possibly due to oxidative stress and evoked vasoconstriction.

